# Survival with Treated and Well-Controlled Blood Pressure: Findings from a Prospective Cohort Study

**DOI:** 10.1371/journal.pone.0017792

**Published:** 2011-04-13

**Authors:** Debbie A. Lawlor, Lois Kim, Richard Morris, Antoinette Amuzu, Peter Whincup, Shah Ebrahim

**Affiliations:** 1 MRC Centre for Causal Analyses in Translational Epidemiology, School of Social and Community Medicine, University of Bristol, Bristol, United Kingdom; 2 Department of Epidemiology and Population Health, London School of Hygiene and Tropical Medicine, London, United Kingdom; 3 Department of Primary Care and Population Health, University College London, London, United Kingdom; 4 Division of Community Health Sciences, St George's, University of London, London, United Kingdom; Innsbruck Medical University, Austria

## Abstract

**Aim:**

To compare survival and incident cardiovascular disease between normotensive, untreated hypertensive, treated and poorly-controlled hypertensive and treated and well-controlled hypertensive adults.

**Methods and Results:**

Data from the British Regional Heart Study (men) and British Women's Heart and Health Study (women) were used (N = 6476). Blood pressure and treatment were assessed at baseline (1998–2001) when participants were aged 60–79 years and participants were followed up for a median of 8 years. Date and cause of death were obtained from death certificates and non-fatal cardiovascular disease events were obtained from repeat detailed medical record reviews. Of the whole cohort 52% of women and 49% of men had untreated hypertension and a further 22% and 18%, respectively, had poorly treated hypertension. Just 3% of women and 4% of men had treated and well controlled hypertension and 23% and 29%, respectively, were normotensive. Compared to normotensive individuals, incident cardiovascular disease (fatal and non-fatal) was increased in those with poorly-controlled hypertension (Hazard Ratio (HR): 1.88; 95%CI: 1.53, 2.30), those with untreated hypertension (HR 1.46; 95%CI 1.22, 1.75) and those who were well-controlled hypertension (HR 1.38; 95%CI 0.94, 2.03). Adjustment for baseline differences in mean blood pressure between the groups resulted in attenuation of the increased risk in the poorly-controlled (1.52 (1.18, 1.97) and untreated groups (1.21 (0.97, 1.52), but did not change the association in the well-controlled group. All-cause mortality was also increased in all three hypertension groups but estimates were imprecise with wide confidence intervals.

**Conclusions:**

Half of women and men aged 60–79 in Britain had untreated hypertension and only a very small proportion of those with diagnosed and treated hypertension were well controlled. Those with hypertension, irrespective of whether this was treated and controlled or not, were at greater risk of future cardiovascular disease than those who are normotensive.

## Introduction

Hypertension and high blood pressure are a major contribution to premature mortality, disease burden and reduced quality of life [1], [2]. High blood pressure and hypertension were responsible for 7.6 million premature deaths and 92 million disability-adjusted life years (DALYs) globally in the year 2001 [2]. Tobacco use, which has a much higher priority for prevention and control, was responsible for 5 million premature deaths in the year 2000 [3].

A large number of randomised controlled trials in different populations have shown that treatment with antihypertensives reduces the risk of all-cause mortality and fatal and non-fatal coronary heart disease (CHD) and stroke, with the magnitudes of these associations being proportional to the extent of blood pressure reduction achieved by treatment [4]–[6]. The effects seen in these trials also reflect the magnitudes of associations of blood pressure with these outcomes in observational epidemiological studies [1]. This might suggest that good control of blood pressure with treatment in those who are hypertensive would see their risk of future cardiovascular disease (CVD) eventually return to that of normotensive individuals. However, in routine primary care, where adherence to treatment and control of blood pressure is likely to be poorer than in trials, how risk compares between normotensives, hypertensives who are treated and well controlled, treated but poorly controlled and untreated is unclear.

One previous study has compared survival in treated and well-controlled hypertensives to that in normotensives [7]. In that study all-cause and CVD mortality over 20 years follow up were greater in 686 hypertensive men who were regularly seen, treated and well-controlled compared to 6810 normotensive men. However, that study was conducted in the 1970s amongst men who were all attending a secondary care outpatient clinic and results may not be generalisable to a contemporary population managed in primary care, where most treatment of hypertension takes place.

The aim of this study is to compare all-cause and cardiovascular mortality and incident cardiovascular disease between normotensive, untreated hypertensive, treated poorly-controlled hypertensive (here after referred to as poorly-controlled) and treated and well-controlled hypertensive (referred to as well-controlled) older adults.

## Methods

### Ethics Statement

UK local and multi-centre medical research ethics committee' approvals were obtained for both studies and participants gave written informed consent for review of their medical records and data collected as part of this study to be used.

### Study participants

Data from two prospective cohort studies - the British Regional Heart Study (BRHS) and the British Women's Heart and Health Study (BWHHS) – collected between 1998 and 2001 using similar protocols were used [8]. The data from BRHS that were used were from 4252 men who were aged 60–79 at the time of baseline assessment of blood pressure used in this study. This data collection represented the 20 year follow-up of survivors in BRHS, which originally comprised 7,735 men who were recruited in 1978–1980 [9]. We only used the data from this 20 year follow-up here for two reasons. First, at this follow-up the BWHHS was initiated and used identical protocols to that used for the BRHS, and therefore we were able to use data on a cohort of older women and men (as opposed only to one of men). Second, less detailed information on hypertensive treatment were available at the original baseline assessment of BRHS participants in 1978–80 and we were interested in how more contemporary antihypertensive treatment options might be associated with survival. For example, ACE-inhibitors were not in widespread use in the 1970s and early 80 s. The BWHHS [10] comprises 4,286 women aged 60–79, who were selected from the same population as the BRHS and first recruited at the time of the re-examination of the BRHS men (i.e. 1998–2001). Both studies used the same design and clinic protocol. After excluding those with pre-existing CHD and stroke at baseline, 3425 women and 3051 men were eligible for inclusion in analyses.

### Assessment of hypertension

For all participants blood pressure and blood pressure treatment were assessed in clinics that took place between 1998 and 2001 when participants were aged 60–79 years. A Dinamap 1846SX vital sign monitor was used to measure blood pressure with correction for systematic over-estimation [11]. Measurements were taken twice in succession with a one-minute interval, with the participant seated, rested and their arm supported at chest level. The mean of the two measurements was used in all analyses.

At the research nurse interview, all participants brought their current medications and gave details of their drug history which were coded to the British National Formulary (BNF) [12].

### Assessment of mortality and CVD events

All participants in both cohort studies have been flagged with the National Health Service Central Register (NHSCR) which provided data on death certificates for participants who died up to 31^st^ December 2007 for BWHHS and 1^st^ June 2007 for BRHS. CVD mortality was defined as an underlying cause of death coded with any of the following international disease code 10 (ICD10) codes: I200–259 (ischeamic heart disease), I516 (cardiovascular disease unspecified), I600–679 (cerebrovascular disease), I690–699 (sequelae of cerebrovascular disease), G450–453 (cranial nerve disorders) or G460–469 (polyneuropathies and other disorders of the peripheral nervous system). Non-fatal CVD events were obtained from medical record reviews that systematically collected information on diagnosed myocardial infarction, angina and stroke every two years.

### Assessment of cardiovascular risk factors

Participants attended the assessment clinic having fasted for a minimum of 8 hours. Participants were instructed to take all medication as normal during this fast and that drinking water was acceptable; those with insulin controlled diabetes were instructed not to fast. Plasma glucose was measured by a glucose oxidase Trinder method [13] using a Hitachi Modular analyser (Hitachi, Kobe, Japan). Serum insulin was measured using an ELISA assay which does not cross react with proinsulin [14]. High density lipoprotein cholesterol (HDL-c) and triglyceride levels were measured using a Hitachi 747 automated analyser (Hitachi, Tokyo, Japan) and reagents supplied by Roche Diagnostics (Basel, Switzerland). Low density lipoprotein cholesterol (LDL-c) was estimated using the Friedwald equation (LDL-c = total cholesterol minus (HDL-c+triglycerides*0.45)) [15]. Standard procedures were used to measure height and weight as previously described [8]. Information on smoking and physical activity was obtained from the baseline questionnaires and research nurse interviews. Childhood and adult social class, based on self-report of fathers and own occupation (for women, substituted with their spouses occupation if own is missing), were classified according to the registrar general (of Britain) classification [16].

### Definitions of hypertension

Participants were categorised on the basis of their baseline blood pressure assessment and antihypertensive treatment into one of four mutually exclusive groups:

1. Normotensive - systolic blood pressure <140 mmHg and diastolic blood pressure <90 mmHg and not on antihypertensive medication;
2. Untreated hypertensive - systolic blood pressure ≥140 mmHg or diastolic blood pressure ≥90 mmHg and not on antihypertensive medication;
3. Treated and poorly-controlled hypertensive - systolic blood pressure ≥140 mmHg or diastolic blood pressure ≥90 mmHg and on antihypertensive medication;
4. Treated and well-controlled hypertensive - systolic blood pressure <140 mmHg and diastolic blood pressure <90 mmHg and on antihypertensive medication.

### Statistical methods

Data were analysed using Cox proportional hazards regression models, with participants' age as the time axis and the date of the baseline assessment (1998–2001) as the start of follow-up for each participant. Contributions to risk were censored at the earlier of: (i) date of death; (ii) date of emigration outside of Britain; (iii) 1^st^ June 2007 and (iv) date of non-fatal CVD event (for the analyses with incidence of non-fatal CVD as the outcome only). Multivariable analyses were adjusted for differences in baseline cardiovascular disease risk factors (body mass index, fasting insulin, glucose and lipids, smoking, physical activity, childhood and adulthood social class) to determine whether these explained any differences in survival between the groups. Fasting glucose, insulin and triglyceride levels were positively skewed and therefore geometric means and 95% confidence intervals are presented for these variables in descriptive analyses and their logged values were used in regression models. Initially, all analyses were conducted separately for women (BWHHS) and men (BRHS). There was no evidence of difference in associations between women and men (all gender interaction p-values>0.1) and therefore analyses used combined data and adjusted for gender. Proportionality assumptions of the Cox models were assessed using Schoenfeld residuals, which showed no evidence of any departure from proportional hazards in the analysis of observed data with no missing covariables.

Of the 3425 eligible women 243 (7%) had missing blood pressure and a further 726 (21%) had missing data on at least one covariable; equivalent numbers for men were 9 (0.3%) and 702 (23%); outcome data were available for all participants. The amount of missing data for any single covariable was small (<10% in all covariables except childhood social class in women (12% missing)). To explore whether missing data might have resulted in selection bias we completed two sets of analyses: one including the 4797 participants (2456 women) with complete data on exposure and all covariables, and one including all eligible participants (6476; 3425 women) with missing exposure or covariable data imputed. We used multiple multivariate imputation, using all variables included in any analyses, the censoring indicator and the log of survival time for Cox models, to impute missing values for those variables with some missing data [17]. We carried out 20 cycles of regression switching and generated 20 imputation datasets separately for men and women. All analyses were conducted using Stata version 11 (Stata corporation, Texas 2005).

## Results

Of the 3182 women with information on blood pressure and treatment, 1641 (52%) had untreated hypertension, 696 (22%) had poorly-controlled hypertension, 101 (3%) well-controlled hypertension and 744 (23%) had normal blood pressure (as noted above 243 (7%) women had missing blood pressure or treatment data). The distribution of blood pressure categories for all 3425 eligible women, including those for whom blood pressure was imputed when it was missing was similar: 54% with untreated hypertension, 20% with poorly-controlled hypertension, 3% with well-controlled hypertension and 23% normotensive. Of the 3042 men 1497 (49%) had untreated hypertension, 545 (18%) had poorly-controlled hypertension, 113 (4%) well-controlled hypertension and 887 (29%) had normal blood pressure (9 (0.3%) had missing blood pressure or treatment data). Thus, for both genders in over 80% of those on treatment, blood pressure was poorly-controlled.

Among the 3425 women, there were 409 deaths over a median follow-up time of 7.6 years, giving a mortality rate of 16.3/1000 person-years. This includes 70 deaths due to cardiovascular disease, giving a cardiovascular mortality rate of 2.8/1000 person-years. A further 325 women experienced a non-fatal cardiovascular disease event, giving a total rate of fatal or non-fatal cardiovascular disease of 16.8/1000 person-years. Among the 3051 men, there were 585 deaths over a median follow-up time of 8.1 years, giving a mortality rate of 25.3/1000 person-years. This includes 95 deaths due to cardiovascular disease, giving a cardiovascular mortality rate of 4.1/1000 person-years. A further 484 men experienced a non-fatal cardiovascular disease event, giving a total rate of fatal or non-fatal cardiovascular disease of 27.2/1000 person-years.

Table 1 shows baseline characteristics by hypertensive group in women and Table 2 the equivalent characteristics in men. In these tables only the 3182 women and 3042 men with complete data on blood pressure and antihypertensive treatment are included. Both women and men with hypertension (irrespective of treatment or control) were older, had larger BMI, higher fasting glucose, insulin and triglycerides than those who were classified as normotensive. Fasting HDLc levels were slightly lower in women and men in the well-controlled categories than other categories. Poorly-controlled hypertensives were more likely to report never taking regular exercise for both women and men, though differences across categories were slight for women. In both women and men those with normal blood pressure were more likely to be current smokers than those in any of the hypertensive categories. Neither childhood nor adult social class were related to hypertensive group in men, and childhood social class was not associated with hypertensive group in women. Women with either untreated or poorly-controlled hypertension were more likely to be from manual social classes in adulthood. As anticipated given the definitions of the groups mean systolic and diastolic blood pressure were highest in those with untreated hypertension and those with treated but poorly-controlled hypertension and lowest in the normotensive and well-controlled group in both genders.

**Table 1:** Baseline characteristics in British women and men aged 60–79 years without cardiovascular disease by categories of hypertension amongst women. N = 3182.

Characteristic	Number with complete data	Mean (SD) for continuous variables or N (%) for binary variables in:				P for heterogeneity across categories*
		Normotensive	Untreated HT	Treated and poorly controlled HT	Treated and well controlled HT	
Number in each category	3182	744	1641	697	101	-
Age (years)	3182	66.3 (5.0)	68.9 (5.4)	69.8 (5.4)	66.8 (5.0)	<0.0005
Systolic BP (mmHg)	3182	124.9 (11.0)	164.7 (18.8)	169.6 (19.1)	129.0 (9.1)	<0.0005$
Diastolic BP (mmHg)	3182	69.7 (7.8)	82.7 (10.5)	84.3 (11.0)	71.7 (7.6)	<0.0005$
Body mass index (kg/m^2^)	3159	26.4 (4.3)	27.2 (4.7)	28.5 (5.2)	29.8 (5.1)	<0.0005$
Fasting glucose**	3055	5.7 (4.3, 7.8)	5.9 (4.2, 8.1)	6.1 (4.1, 9.2)	6.0 (3.7, 9.6)	<0.0005$
Fasting Insulin**	3086	5.9 (1.8, 19.6)	6.7 (1.9, 23.2)	7.6 (2.0, 29.2)	8.6 (1.9, 38.2)	<0.0005$
Fasting HDLc	3067	1.7 (0.4)	1.7 (0.5)	1.6 (0.4)	1.5 (0.3)	<0.0005
Fasting LDLc	3002	4.1 (1.0)	4.3 (1.1)	4.2 (1.1)	4.1 (1.1)	0.003
Fasting Triglyceride**	3073	1.5 (0.6, 3.4)	1.6 (0.7, 4.0)	1.9 (0.8, 4.6)	1.7 (0.8, 3.9)	<0.0005
Current smoker (%)†	3180	112 (15%)	186 (11%)	49 (7%)	8 (8%)	<0.0005
No regular exercise (%)	3082	23 (3%)	69 (4%)	43 (6%)	5 (5%)	0.05
Manual childhood social class (%)$$ †	2828	489 (74%)	1105 (76%)	474 (77%)	75 (82%)	0.2
Manual adult social class (%)$$ †	2905	202 (30%)	535 (35%)	235 (38%)	27 (30%)	0.02
On ≥2 blood-pressure drugs (%)	N/A***	-	-	218 (31%)	32 (32%)	-

In this table results are only presented for those with complete data, and for those with non-missing hypertensive status; HT = Hypertensive.

*i.e. the p-value tests whether the distribution differs between any of the groups and has 3 degrees of freedom.

**For these variables geometric means and 95% confidence intervals are presented.

$ Using robust standard errors to account for differences in variances between groups.

$$ Manual social class defined as social class III manual/class IV/class V/armed forces.

† Included as categorical covariable with all levels (never/ex/current smoker, all levels of social class) in analysis.

***Response not required if not on drugs.

**Table 2:** Baseline characteristics in British women and men aged 60–79 years without cardiovascular disease by categories of hypertension amongst men. N = 3042.

Characteristic	N complete data	Mean (SD) for continuous variables or N (%) for binary variables in:				P for heterogeneity across categories*
		Normotensive	Untreated HT	Treated and poorly controlled HT	Treated and well controlled HT	
Number in each category	3042	887	1497	545	113	-
Age (years)	3042	67.0 (5.1)	68.5 (5.5)	69.3 (5.3)	68.4 (5.3)	<0.0005
Systolic BP (mmHg)	3042	124.6 (10.9)	160.9 (17.6)	165.9 (18.9)	125.9 (11.5)	<0.0005$
Diastolic BP (mmHg)	3042	77.2 (7.0)	89.9 (10.0)	91.3 (10.1)	78.8 (6.0)	<0.0005$
Body mass index (kg/m^2^)	3036	26.1 (3.6)	26.7 (3.5)	27.7 (3.5)	28.2 (4.2)	<0.0005$
Fasting glucose*	2906	5.6 (4.1, 7.8)	5.8 (3.9, 8.6)	6.1 (3.8, 9.8)	6.2 (3.6, 10.8)	<0.0005$
Fasting Insulin*	2895	7.4 (2.2, 24.7)	7.9 (2.3, 26.6)	9.0 (2.5, 32.7)	9.7 (2.8, 33.9)	<0.0005$
Fasting HDLc	2886	1.3 (0.2)	1.4 (0.4)	1.3 (0.3)	1.2 (0.3)	<0.0005$
Fasting LDLc	2865	3.9 (0.9)	4.0 (1.0)	3.9 (1.0)	3.9 (1.0)	<0.0005
Fasting Triglyceride*	2903	1.5 (0.6, 3.9)	1.6 (0.6, 4.1)	1.7 (0.7, 4.5)	1.8 (0.6, 4.9)	<0.0005
Current smoker (%)†	2836	183 (22%)	266 (19%)	88 (18%)	20 (19%)	0.2
No regular exercise (%)	2972	413 (48%)	717 (49%)	314 (59%)	46 (42%)	<0.0005
Manual childhood social class (%)$$ †	2753	621 (78%)	1089 (80%)	401 (81%)	88 (82%)	0.5
Manual adult social class (%)$$ †	3035	462 (52%)	774 (52%)	290 (53%)	57 (51%)	0.9
On ≥2 blood-pressure drugs (%)	N/A***	-	-	173 (32%)	28 (25%)	-
Years on blood-pressure lowering drugs (median (IQR))	N/A	N/A	N/A	6 (0,7)	3 (0,7)	-

In this table results are only presented for those with complete data, and for those with non-missing hypertensive status; HT = Hypertensive.

*i.e. the p-value tests whether the distribution differs between any of the groups and has 3 degrees of freedom.

**For these variables geometric means and 95% confidence intervals are presented.

$ Using robust standard errors to account for differences in variances between groups.

$$ Manual social class defined as social class III manual/class IV/class V/armed forces.

† Included as categorical covariable with all levels (never/ex/current smoker, all levels of social class) in analysis.

***Response not required if not on drugs.

Table 3 shows all-cause mortality by hypertensive group. The results based on participants with complete data only and those with all eligible participants (with imputed data) were generally similar. The risk of death for all participants with hypertension (irrespective of whether treated and, if so, whether well controlled) was greater than normotensive individuals. Risk seemed to be highest for those who were treated and well controlled, but the numbers in this category were small and there was no statistical evidence that the hazard ratio differed for any of the three subgroups of individuals with hypertension (p-values for differences in coefficients derived from 1000 bootstrap replications to estimate standard errors of differences between log hazard all >0.3). Adjustment for covariables (second model) or additional adjustment for differences in mean blood pressure across the categories (third model) did not markedly alter the associations.

**Table 3:** Association of hypertension categories with all-cause mortality in women and men combined.

		Hypertensive group			
	Total	Normotensive	Untreated hypertensive	Poorly treated hypertensive	Well treated hypertensive
***Using subset with no missing values for any covariable/hypertensive group***					
N	4796	1264	2427	941	164
Deaths	635	130	339	139	27
Hazard ratios (95% CI)					
Model 1*	-	1	1.14 (0.93, 1.40)	1.17 (0.92, 1.49)	1.48 (0.98, 2.25)
Model 2*	-	1	1.17 (0.95, 1.44)	1.22 (0.95, 1.56)	1.48 (0.97, 2.25)
Model 3*	-	1	1.20 (0.92, 1.57)	1.25 (0.91, 1.72)	1.47 (0.97, 2.24)
***Using imputed data for missing values (20 imputations)***					
N**	6476	1688	3328	1245	215
Deaths**	950	198	519	199	34
Hazard ratios (95% CI)					
Model 1*	-	1	1.15 (0.96, 1.36)	1.12 (0.92, 1.37)	1.26 (0.87, 1.81)
Model 2*	-	1	1.19 (1.00, 1.41)	1.17 (0.95, 1.44)	1.30 (0.90, 1.88)
Model 3*	-	1	1.15 (0.92, 1.44)	1.13 (0.87, 1.46)	1.28 (0.89, 1.85)

*Model 1: adjusted for sex, age (as time variable in Cox model).

Model 2: additionally adjusted for BMI, smoking, physical activity, LDL cholesterol, HDL cholesterol, triglycerides (log scale), glucose (log scale), insulin (log scale), fathers occupational social class (childhood social class), own adult occupational social class.

Model 3: additionally adjusted for systolic and diastolic blood pressure (BP).

**Imputed numbers per group and associated numbers of events are means over 20 imputed datasets.

Numbers per group differ slightly between Tables 2, 3, 4 as time to event and the censoring variable are included as a covariable in the imputation prediction model.

Table 4 shows cardiovascular mortality by hypertensive group. Results were similar for the complete dataset analysis and the imputed dataset analysis. Participants with hypertension, irrespective of treatment or whether controlled, had around double the risk of cardiovascular mortality over the follow-up period with no evidence that this increased risk differed by whether the patient was treated or not or whether they were well-controlled (if treated) or not (p-values for differences in coefficients derived from 1000 bootstrap replications to estimate standard errors of differences between log hazard all >0.5). Adjustment for potential covariables did not importantly alter these associations (second model). However, additional adjustment for baseline differences between the groups in systolic and diastolic blood pressure (third model) attenuated the increased risk in untreated and poorly treated hypertensives compared with normotensives towards the null, whereas the increased risk of cardiovascular mortality in the treated and well controlled group remained largely unchanged.

**Table 4:** Association of hypertension categories with CVD mortality in women and men combined.

		Hypertensive group			
	Total	Normotensive	Untreated hypertensive	Poorly treated hypertensive	Well treated hypertensive
***Using subset with no missing values for any covariable/hypertensive group***					
N	4796	1264	2427	941	164
CVD deaths	107	15	59	29	4
Hazard ratios (95% CI)					
Model 1*	-	1	1.60 (0.90, 2.85)	1.98 (1.05, 3.72)	1.85 (0.61, 5.58)
Model 2*	-	1	1.66 (0.93, 2.96)	2.09 (1.09, 4.00)	1.70 (0.56, 5.22)
Model 3*	-	1	1.09 (0.54, 2.20)	1.29 (0.58, 2.89)	1.67 (0.55, 5.12)
***Using imputed data for missing values (20 imputations)***					
N**	6476	1688	3328	1245	215
CVD deaths**	158	21	92	39	6
Hazard ratios (95% CI)					
Model 1*	-	1	1.80 (1.10, 2.94)	1.97 (1.14, 3.39)	2.04 (0.82, 5.08)
Model 2*	-	1	1.91 (1.17, 3.12)	2.13 (1.23, 3.71)	1.97 (0.78, 4.94)
Model 3*	-	1	1.19 (0.65, 2.18)	1.26 (0.64, 2.47)	1.92 (0.77, 4.83)

*Model 1: adjusted for sex, age (as time variable in Cox model).

Model 2: additionally adjusted for BMI, smoking, physical activity, LDL cholesterol, HDL cholesterol, triglycerides (log scale), glucose (log scale), insulin (log scale), fathers occupational social class (childhood social class), own adult occupational social class.

Model 3: additionally adjusted for systolic and diastolic blood pressure (BP).

**Imputed numbers per group and associated numbers of events are means over 20 imputed datasets.

Numbers per group differ slightly between Tables 2, 3, 4 as time to event and the censoring variable are included as a covariable in the imputation prediction model.

Table 5 shows all cardiovascular events (fatal plus non-fatal) by hypertensive group in women and men combined. These results were generally consistent with those for CVD mortality, with the exception that in model 3 (adjustment for baseline differences in systolic and diastolic blood pressure) the attenuation in those who were poorly treated was somewhat less marked than in the analyses with fatal outcomes only.

**Table 5:** Association of hypertension categories with fatal and non-fatal CVD in women and men combined.

		Hypertensive group			
	Total	Normotensive	Untreated hypertensive	Poorly treated hypertensive	Well treated hypertensive
***Using subset with no missing values for any covariable/hypertensive group***					
N	4793	1263	2426	941	164
Events	698	142	354	180	22
Hazard ratios (95% CI)					
Model 1*	-	1	1.32 (1.08, 1.60)	1.77 (1.41, 2.21)	1.25 (0.80, 1.95)
Model 2*	-	1	1.29 (1.06, 1.57)	1.73 (1.37, 2.18)	1.10 (0.70, 1.73)
Model 3**	-	1	1.08 (0.84, 1.40)	1.42 (1.06, 1.90)	1.09 (0.69, 1.71)
***Using imputed data for missing values (20 imputations)***					
N**	6473	1692	3322	1244	215
Events**	956	177	504	243	32
Hazard ratios (95% CI)					
Model 1*	-	1	1.47 (1.23, 1.75)	1.92 (1.58, 2.34)	1.47 (1.01, 2.15)
Model 2*	-	1	1.46 (1.22, 1.75)	1.88 (1.53, 2.30)	1.38 (0.94, 2.03)
Model 3*	-	1	1.22 (0.97, 1.52)	1.52 (1.18, 1.97)	1.38 (0.94, 2.03)

*Model 1: adjusted for sex, age (as time variable in Cox model).

Model 2: additionally adjusted for BMI, smoking, physical activity, LDL cholesterol, HDL cholesterol, triglycerides (log scale), glucose (log scale), insulin (log scale), fathers occupational social class (childhood social class), own adult occupational social class.

Model 3: additionally adjusted for systolic and diastolic blood pressure (BP).

**Imputed numbers per group and associated numbers of events are means over 20 imputed datasets.

Numbers per group differ slightly between Tables 2, 3, 4 as time to event and the censoring variable are included as a covariable in the imputation prediction model.

In men only we had information on duration of antihypertensive treatment (shown in Table 2). In the men we therefore examined the association between duration of treatment and outcomes. With adjustment for all potential confounding factors (equivalent to Model 2 in previous tables) the hazard ratio of all-cause mortality for each additional year of treatment was 1.01 (95%CI: 0.98, 1.04) and that for cardiovascular disease (fatal and non-fatal combined) was 1.00 (95% CI 0.97, 1.03) indicating no impact of duration of treatment. Additional adjustment for baseline blood pressure (equivalent to model 3) did not change either of these results.

Despite a large sample size (6476) and 8 years of follow-up we found that we had limited statistical power to determine whether those who are well controlled on treatment had similar risks to those who were normotensive at baseline; largely due to the very small proportion who were treated and well-controlled. The sample size that would be required to demonstrate non-inferior survival compared to the normotensive group (i.e. a return in those with well controlled hypertension to similar levels as those who were normotensive) is over 40,000 participants. This is based on calculations assuming the same ratio of normotensives to well controlled hypertensives and the same incidence of CVD for normotensives as in our study, a maximum clinically irrelevant hazard ratio of 1.1, and Type I and Type II errors of 10%.

## Discussion

It is notable that in this cohort of 60–79 year British men and women, who were initially examined between 1998 and 2001, around half were found to have untreated hypertension. A further 25% of women and 22% of men were being treated for hypertension but the majority of these (over 80% in both genders) were poorly controlled (ie. >140/90 mmHg). Using the British Hypertension Society treatment targets, (≤140/85 mmHg) the proportion who were well controlled on treatment decreased slightly from 113 (3.7%) to 94 (3.1%) in women and from 101 (3.0%) to 96 (2.8%) in men.

Women and men with hypertension (irrespective of whether they were on treatment or if so whether their blood pressure was well controlled) had increased risk of all cause and CVD mortality. With respect to CVD mortality (and to a lesser degree fatal and non-fatal CVD), the increased risk in those with untreated hypertension and treated but poorly controlled hypertension was importantly influenced by differences in mean baseline blood pressure between these groups and those who were normotensive, with substantial attenuation to the null once this was adjusted for in multivariable models. However, the elevated risk in the well-controlled group compared to the normotensive group was not explained by baseline differences in blood pressure. Our finding of increased risk of all-cause and cardiovascular disease mortality in those who were on treatment and well controlled is consistent with one previous study of men only [7]. That study had considerably greater numbers in the well treated and controlled group (and hence greater power) than in our study since these were specifically selected from a clinic that monitored this group regularly.

It is clear from randomised controlled trials that treatment and good control of hypertension reduces the risk of CVD events and increases survival compared to no treatment [4]–[6]. In these trials stroke risk appears to fall within 2 or 3 years to normotensives levels whereas only half the expected reduction on treatment is seen for CHD risk [18]. There could be several reasons why we find similar increased risk in those who are well-controlled to those who are untreated and those treated but not well-controlled, and indeed greater risk of CVD once differences for baseline blood pressure are taken into account. First, it is possible that participants in the treated and well controlled group included a substantial proportion who had only been treated for a relatively short period of time but in men, at least, we found no evidence that duration of treatment was associated with risk of all-cause mortality or CVD. It is possible that dose of treatment is more relevant that duration, or that those in the treated and well-controlled group are adhering to their treatment more so than those in the poorly-controlled group, but we are unable to explore this. Second, we do not have time updated measurements of blood pressure in this cohort and it is possible that those who were in the well controlled group became poorly controlled over the follow-up period. Third, it is possible that good control on treatment reflects more aggressive treatment because these were already identified by doctors as more at risk patients. Fourth, cardiovascular risk is multi-factorial and here we focused on high blood pressure but adjustment for other CVD risk factors did not have a marked impact on our findings. Fifth, we did not have sufficient power to rule out a clinically important treatment effect, estimating that we would need a sample size of at least 40,000 to demonstrate (with high levels of precision) no difference in survival or CVD between the well controlled group and the normotensive group over 10 years of follow-up. Lastly, it may be that for an important proportion of those with poorly controlled hypertension, blood pressure has been elevated for a long period prior to their initial diagnosis and then subsequent treatment. The substantial number in our cohort with untreated hypertension lends support to this suggestion. As a consequence of prolonged untreated elevated blood pressure, irreversible vascular damage may have occurred, and whilst treatment will reduce risk compared to no treatment in this scenario, it may be unreasonable to expect this risk to be decreased to levels seen in those who have always been normotensive.

As our results, and those of other studies [19], [20], highlight that a substantial proportion of individuals with hypertension remain undiagnosed and of those who are diagnosed a substantial proportion remain untreated and/or poorly controlled, there is still a need for better surveillance to identify, treat and monitor those with hypertension. The baseline data for this study were collected just prior to publication and implementation of the UK National Service Framework [21]. They also predate the 2004 UK New General Practitioner Contract, which specified in detail the services to be provided in primary care and began paying practices of achieving specified targets [22]. General practitioners meeting the quality of clinical care required 19% of the total maximum possible payment provided by the new contract [22]. Thus, the introduction of this contract may have improved identification and treatment of hypertension. A recent evaluation of this scheme focused on just three chronic diseases – coronary heart disease, asthma and diabetes – and did not include hypertension [23].

### Study strengths and limitations

The main strength of this study is that it addresses an important clinical question that has only been addressed previously in one published study that only included men who were attending secondary care clinics for treatment of their hypertension. The population are a random sample of women and men from across the UK. However, we cannot assume that our results would be generalisable to a younger population. An important limitation is the lack of time updated information on blood pressure and antihypertensive medication, and further study with repeat measurements of these would be valuable. It is possible that those who were well-controlled at baseline became less so over time and those who were poorly controlled became more so with these two opposite changes resulting in similar risk of outcomes in these two groups. Thus, our findings should be interpreted as representing differences in risk over an eight-year follow-up period amongst groups of women and men whose hypertensive status was defined at the start of that period only. Blood pressure was measured twice for each participant and the mean of these two measurements used, but both measurements were at one clinic setting and whilst this is consistent with how hypertension is used in epidemiological research, in clinical practice a diagnosis of hypertension would require a second elevated measurement on a separate occasion. In addition we have no information in this study on adherence to prescribed treatment. We know that the participants stated that they were taking their medication but we did not check this in any formal way. The male participants (BRHS) in this cohort consisted of survivors from a cohort that started 20 years prior to the assessment that we used as baseline here. This could have resulted in some selection (survivor) bias. However, we found no evidence that findings in men differed from those in the BWHHS women, who were newly recruited at the time of baseline assessment for this study. An additional limitation is that the very small proportion of those with hypertension who are treated and well-controlled means that we have limited statistical power to precisely estimate risk in this group. As is common in epidemiological studies we used two measures of blood pressure taken at the same clinic, whereas hypertension in clinical practice is based on repeat measurements on different days over a three-month period. Thus, our estimate of untreated hypertensives may overestimate what would be found in clinical practice.

### Conclusions

Whilst treatment of hypertension clearly reduces risk of CVD and improves survival compared to no treatment [4]–[6], our results, and those of one previously published study [7], suggest that individuals in non-trial routine clinical practice with hypertension who are treated and well-controlled remain at increased risk of all-cause mortality and CVD compared to individuals who are normotensive. Our results also highlight the high proportion of individuals with untreated hypertension and the high proportion of those treated who are poorly controlled. These findings emphasise the importance of prevention of hypertension and continued monitoring of those on treatment with modifications to achieve good control. Given the small proportion in our study who were well-controlled further examination of this question in large cohort studies is required. Large scale routine general practice data bases (e.g. QRESEARCH database) would be capable of answering the important question of whether survival and cardiovascular disease risk in well-controlled hypertensives reverts to that seen in normotensives over a reasonable follow-up period.
